# Exploring Facility Revisit Intentions Among the Kidney Dialysis Patient’s Attendance: Evidence from a Cross-Sectional Study in Dhaka, Bangladesh

**DOI:** 10.3390/ijerph23060769

**Published:** 2026-06-07

**Authors:** Tanvir Fittin Abir, Rakibul Islam, Kazi Fayzus Salahin, Kaniz Kakon, Kingsley Emwinyore Agho, Sandy Francis Peris, Khan Sarfaraz Ali

**Affiliations:** 1 Department of Business Administration, Faculty of Business and Entrepreneurship, Daffodil International University, Ras Al Khaimah Campus, Ras Al Khaimah P.O. Box 10021, United Arab Emirates; campusdirector@daffodiluniversity.ae; 2 Department of Business Administration, Faculty of Business and Entrepreneurship, Daffodil International University, Dhaka P.O. Box 1216, Bangladesh; islam14-458@diu.edu.bd; 3 PSU Center for Global Health Research and Innovation (C-GHRi), Faculty of Medicine, Prince of Songkla University, Hat Yai, Songkhla 90110, Thailand; 4 Department of Epidemiology, Faculty of Medicine, Prince of Songkla University, Hat Yai, Songkhla 90110, Thailand; 5 Department of Philosophy, College of Arts and Sciences (CAAS), International University of Business, Agriculture and Technology, Dhaka 1230, Bangladesh; kanizkakon@iubat.edu; 6 School of Medicine, Faculty of Health, Western Sydney University, Locked Bag 1797, Penrith, NSW 2571, Australia; k.agho@westernsydney.edu.au; 7 Department of Business Studies, State University of Bangladesh, Dhaka 1461, Bangladesh; perisandy@gmail.com; 8 Faculty of Business and Communications (FBC), INTI International University, Nila 71800, Malaysia; khan.sarfarazali@newinti.edu.my

**Keywords:** revisit intention, patient satisfaction, dialysis service quality, healthcare trust, urban health, good health and well-being, Bangladesh

## Abstract

**Highlights:**

**Public health relevance—How does this work relate to a public health issue?**

- Chronic kidney disease is a growing burden in Bangladesh, with limited urban focused-dialysis services available.
- Understanding revisit intention among the patient’s attendance insight into continuity of care in urban health systems.

**Public health significance—Why is this work of significance to public health?**

- The study identifies that the key determinants of revisit behavior are cost, perceived trust in healthcare providers, and patient satisfaction.
- Findings also highlight the mediating role of satisfaction in linking service quality, trust, and affordability to patient loyalty.

**Public health implications—What are the key implications or messages for practitioners, policy makers and/or researchers in public health?**

- Improving affordability and strengthening perceived trust in healthcare providers can enhance patient retention in dialysis services.
- Providers and policymakers should prioritize patient-centered strategies to improve satisfaction and ensure sustainable dialysis care in urban Bangladesh.

**Abstract:**

Chronic kidney disease (CKD) is a rising public health concern in low- and middle-income countries (LMICs), with urban populations disproportionately affected. In Bangladesh, particularly in Dhaka, dialysis services have become essential for CKD management. This study investigates the determinants of revisit intention among adult attendants of dialysis patients in Dhaka, using partial least squares structural equation modeling. A cross-sectional survey was conducted across four major dialysis centers totaling 399 valid responses. A purposive sampling technique was employed to ensure the inclusion of respondents with relevant experience and engagement in dialysis service utilization. Among respondents, over half were male, 43% had primary to higher secondary education, and one-third reported household incomes between BDT 40,001 and 60,000. The largest age group was 45–49 years (32.3%), and nearly 60% selected the facility due to nearness. Reliability and validity metrics met recommended thresholds, and multivariate normality was not assumed (Mardia’s test, *p* < 0.05). The structural model revealed significant direct effects of cost (β = 0.167, *p* = 0.003), Perceived trust in healthcare providers (β = 0.252, *p* < 0.001), and Perceived patient satisfaction (β = 0.422, *p* < 0.001) on Perceived revisit intention. Dialysis Delivery Service and word of mouth influenced revisit behavior indirectly through Perceived patient satisfaction. All mediation paths were statistically significant and classified as complementary. To improve patient retention, the policymaker should prioritize affordability, perceived trust in healthcare providers, and overall service quality, which together enhance perceived patients’ satisfaction and revisit intention.

## 1. Introduction

Chronic kidney disease (CKD) represents an escalating global public health challenge, with urban populations in low- and middle-income countries (LMICs) experiencing a disproportionately high disease burden. In Bangladesh, particularly in Dhaka the capital city, the rising prevalence of CKD has rendered dialysis services indispensable to chronic disease management. Contributing factors such as lifestyle transitions, increasing incidences of diabetes and hypertension, and limited access to preventive care have accelerated this trend [1,2]. Within the jurisdictions of the Dhaka North City Corporation (DNCC) and Dhaka South City Corporation (DSCC), dialysis centers serve a rapidly expanding patient base, underscoring the urgent need for sustained improvements in service quality, affordability, and patient-centered care [3,4,5].

In this context, understanding the determinants of revisit intention, the likelihood that patients or their attendants will return to the same dialysis facility for subsequent treatments, has become critically important. Revisiting intention functions as a key behavioral indicator of healthcare quality, satisfaction, and loyalty, offering valuable insight into service performance and continuity of care. Prior studies have identified several constructs shaping revisit behavior, notably Dialysis Delivery Service Quality (DDS), trust in healthcare providers (THP), word of mouth (WOM), and cost (CO) [6,7,8,9]. These factors interact in complex ways to influence patient decision-making, particularly within resource-constrained urban health systems.

Service quality is often conceptualized through dimensions of reliability, responsiveness, and empathy and has consistently been linked to patient satisfaction and long-term retention [10,11,12,13]. Likewise, trust in healthcare providers, cultivated through effective communication, perceived competence, and ethical conduct, is fundamental to adherence and sustained care engagement [14,15,16]. Word of mouth, reflecting patients’ informal evaluations of perceived value, further shapes reputation and facility choice, while financial cost remains a decisive barrier influencing access and continuity [17,18,19,20]. In healthcare, this connection goes deeper: patients who feel cared for and respected are more likely to trust their providers and recommend them to others. While the financial cost remains a barrier, it directly affects SDG 3 (Good Health and Well-being) by limiting equitable access to quality healthcare.

Patient satisfaction (PS) is widely recognized as a mediating construct linking service quality, trust, word of mouth, and cost to revisit intention [21,22,23]. Additionally, disease severity or stage may moderate these associations, as patients at more advanced stages of CKD typically have greater dependency on reliable, trustworthy, and affordable care [6,24,25]. While the relationship among the variables and the revisit intention are well established, this study applies and tries to explore the under-researched context of the dialysis care in Bangladesh. The dialysis is primarily financed out-of-pocket, but cost varies based on the disease’s magnetite and facility types, as the private facilities charged higher compared to public facilities.

Despite the growing relevance of these behavioral and perceptual factors, empirical research examining their integrated influence on revisit intention remains scarce in the Bangladeshi dialysis context. Addressing this critical gap, the present study explores revisit behavior among adult attendants of dialysis patients in Dhaka using partial least square structural equation modeling (PLS-SEM) to evaluate hypothesized relationships. Specifically, the study aims to: (1) assess the level of revisit intention among adult attendants of dialysis patients in Dhaka, (2) identify and evaluate the key determinants influencing revisit intention through PLS-SEM, and (3) validate the measurement model and assess the structural relationships among latent constructs associated with revisit behavior. By elucidating these relationships, the study seeks to generate actionable insights to guide policymakers and healthcare managers in enhancing dialysis service quality, strengthening patient satisfaction and trust, and fostering long-term loyalty within Bangladesh’s urban healthcare landscape.

## 2. Materials and Methods

### 2.1. Study Design and Participants

We conducted a facility-based cross-sectional study in Dhaka, Bangladesh, between September 2024 and January 2025. The study targeted the adult (≥18 year) individuals’ attendants with the patients currently undergoing dialysis treatment. We excluded attendants of the patients with severe medical conditions, those admitted to inpatient care, and individuals unwilling to participate. However, we selected the attendants because of their role in managing costs, logistics, support and facility choice with the cultural context and service utilization decision.

### 2.2. Sample Size and Sampling

The sample size of the study was calculated using the single proportion formula, assuming 50% of the respondents would express to revisit the same institutions for dialysis facility. With a 95% confidence level and 5% margin of errors, the initial sample was estimated at 384. To account for a 10% non-response rate, the adjusted sample size became approximately 426. However, for the final analysis we considered 399 samples after removing the incomplete responses. We purposively selected the four hospitals; namely Bangladesh Specialized Hospital Limited (BSH), Dhanmondi General and Kidney Hospital Limited (DGKHL), Kidney Foundation Bangladesh (KFB) and Sir Salimullah Medical College Mitford Hospital (SMCMH) for data collection. We selected these facilities purposively to ensure inclusion of facilities with high patients’ volume and diverse proprietorship.

### 2.3. Data Collection

We collected data through the exit-interviews conducted in the selected facilities. The distribution of the sample of each facility was as follows: 119 patients from KFB, 76 patients from BSH, 98 patients from DGKHL, and 106 patients from SMCMH. Participants completed a self-administered questionnaire, with trained research staff available to explain the study objectives and clarify any questions. Written informed consent was obtained from all participants prior to data collection.

### 2.4. Tools Development

The survey instrument was developed based on existing literature and was adapted and modified to suit the study context (see Table S1) [9,26,27,28]. The questionnaire was comprised of two main sections: (1) general demographic of the respondents, consisting of 9 questions; and (2) patient intends to revisit the dialysis facility, consisting of 26 questions. The intent-to-revisit section included five sub-domains, each assessed using a 5-point Likert scale ranging from 1 (“Strongly Disagree”) to 5 (“Strongly Agree”) and were coded numerically for analysis. The instrument was localized using a back-translation method to ensure linguistic and cultural appropriateness. In addition, income, education, age, and proximity were considered as contextual covariates to interpret revisit behavior. All items were responded by the attendants or primary caregivers based on their perception and observation of dialysis related to healthcare experience, provider interaction and treatment process.

### 2.5. Statistical Analysis

We use descriptive statistics to show the respondents characteristics using percentage and frequency and partial least square structural equation modeling (PLS-SEM) for analysis of the complex models to determine the patient’s intent to visit with other latent variables. The model evaluation included the key metrics such as the coefficient of determination (R^2^), predictive relevance (Q2), and effect size (f2) [29]. For additional robustness analyses, we consider bootstrap Confidence Interval, PLSpredict assessment, and multi-group analysis (MGA), to evaluate the predictive performance and stability of the model across public and private dialysis facilities. We chose the PLS-SEM because it is a distribution-free approach suitable for predictive modeling and analysis of complex models with multiple mediation paths [30]. To test it we use the Mardia’s multivariate co-efficient having a *p*-value below 0.05 [31], indicating that the data did not meet the assumption of the multivariate normality, further supporting use of the PLS-SEM over covariance-based SEM (CB_SEM). The data was analyzed using SmartPLS v3.4.1 and R v4.5.1.

### 2.6. Ethical Considerations

The study was conducted in accordance with the Declaration of Helsinki. All participants were informed of the purpose of the research, their rights as participants, and the confidentiality of their responses. Written informed consent was obtained from all participants prior to data collection, and they were assured that their participation was voluntary. Ethical approval was obtained from the Daffodil International University bioethics committee (Ref: DIU/DoR/EC/240103).

### 2.7. Hypothesis Development and Conceptual Model

Based on the SERVQUAL framework, reliability, responsiveness, tangibility, and assurance are the most important determinants of patients’ satisfaction and behavioral intentions [9,10]. In healthcare settings such as dialysis care, affordability and perceived service quality play important roles in shaping patients’ experiences and access to care. Previous research has shown that positive perceptions of service quality and affordability are associated with greater patient satisfaction and increased likelihood of returning to the healthcare facility for future care [32]. Cost is a key factor in shaping both satisfaction and perceived revisit intention, especially in resource-constrained healthcare settings. When attendants perceive dialysis services as affordable and reasonable, they are more likely to report positive experiences and continue using the same facility [3]. Based on this, we propose hypothesis (H)1: Cost has a positive effect on perceived patient satisfaction and H5: Cost has a positive effect on perceived revisit intention.

Dialysis service quality including cleanliness, staff responsiveness, and technical care can influence satisfaction levels. A study found that service quality significantly predicted satisfaction in community health centers [33]. However, its direct effect on perceived revisit intention may vary depending on other factors. Therefore, we hypothesize H2: Dialysis service quality has a positive effect on perceived patient satisfaction and H6: Dialysis service quality has a positive effect on perceived revisit intention.

In the organizational trust theory, [34] conceptualized trust as confidence in the competence, integrity, and benevolence of service providers. Healthcare providers are no exception to these. The proposed relationship between trust and patient outcomes is grounded based on that. Perceived trust in healthcare providers is another important factor. When attendants feel confident in the competence and care of doctors and nurses, they are more likely to be satisfied and return to the same facility [35]. Thus, we propose H3: Perceived trust in healthcare providers has a positive effect on perceived patient satisfaction and H8: Perceived trust in healthcare providers has a positive effect on perceived revisit intention.

Word of mouth such as recommendations from peers or community members can shape expectations and perceptions of care. While it may influence satisfaction, its direct impact on perceived revisit intention is less consistent [3]. Based on this, we propose H4: Word of mouth has a positive effect on patient satisfaction and H9: Word of mouth has a positive effect on perceived revisit intention.

The relationship between perceived patient satisfaction and revisit intention is framed by Theory of Planned Behavior (TPB). Ref. [36] suggests that positive experiences and attitudes influence individuals’ future behavioral intentions. In healthcare settings, satisfied patients and attendants are more likely to continue utilizing the same healthcare facility for ongoing treatment and care.

**Figure 1:**
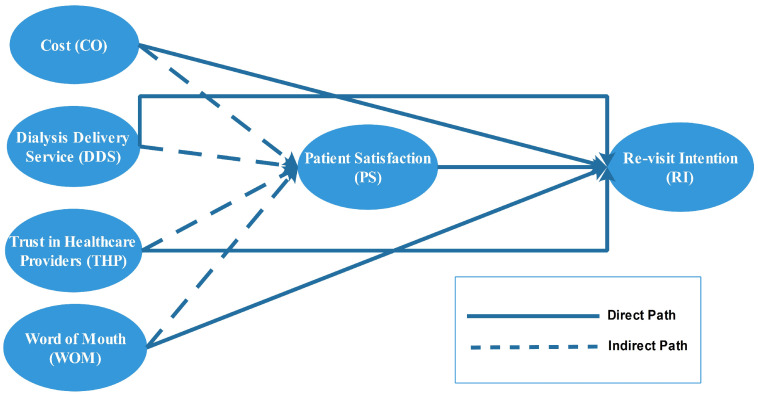
Conceptual Model.

Finally, perceived patient satisfaction itself is a strong predictor of revisit behavior. Satisfied attendants are more likely to return to the same facility for ongoing care [35]. Therefore, we propose H7: Perceived patient satisfaction has a positive effect on perceived revisit intention. We developed the hypothesis based on SERVQUAL framework, organizational trust theory, and the Theory of Planned Behavior, to collectively explain how perceived healthcare quality, trust and the patients and their attendant experiences influence the satisfaction and shape the revisit intention. Based on the hypothesis, we developed our conceptual model for our research, presented in Figure 1.

## 3. Results

### 3.1. Socio-Demographic Characteristics of the Respondents

The socio-demographic characteristics of the 399 respondents were presented in Table 1. Over half of the respondents were male, and approximately 43% had attained education at the primary, secondary, or higher secondary level. One-third of the respondents reported a monthly household income between BDT 40,001 and 60,000, with the largest proportion falling within the 45–49 age group. The majority resided in urban areas, and nearly 60% selected the dialysis facility due to its proximity to their household.

### 3.2. Reliability

All constructs demonstrated acceptable internal consistency, with Cronbach’s Alpha values ranging from 0.700 to 0.796. The composite eliability values were also satisfactory, ranging from 0.815 to 0.863 and the Average Variance Extracted values for all constructs were above the 0.50 benchmark, confirming convergent validity. Multicollinearity and common method bias were assessed using Variance Inflation Factor (VIF). The VIF values were ranging from 1.27 to 1.88 which were well below the recommended threshold of 3.3 [29,37], suggesting no serious collinearity concerns among the predictors and there was no threat of validity of the findings. Reliability of the constructs is presented in Table S2.

### 3.3. Validity

Discriminant validity was established using both the Fornell-Larcker criterion and the Heterotrait-Monotrait (HTMT) ratio. Each construct’s square root of AVE exceeded its correlations with other constructs, and HTMT values remained below the conservative threshold of 0.85, confirming adequate discriminant validity across all latent variables. Discriminant validity is presented in Table S3 [29,38,39].

### 3.4. Structural Model

**Figure 2:**
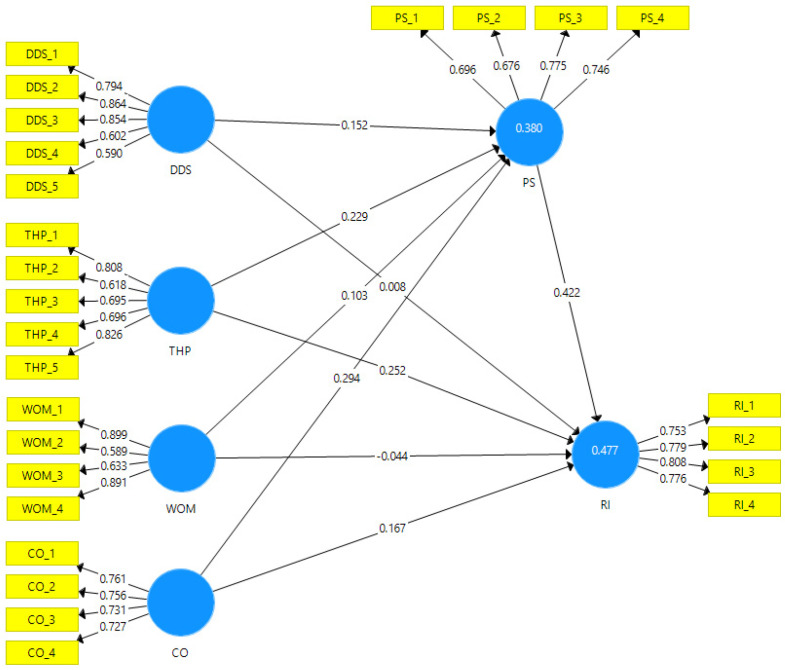
Path Model.

The structural model results, summarized in Table 2, revealed several significant relationships. Cost (CO), Dialysis Delivery Service (DDS), perceived trust in healthcare providers (THP), and word of mouth (WOM) all had significant positive effects on perceived patient satisfaction (PS), with path coefficients ranging from 0.103 to 0.294. In terms of direct effects on revisit intention (RI), CO (β = 0.167, *p* = 0.003) had a significant effect, THP (β = 0.252, *p* < 0.001), and PS (β = 0.422, *p* < 0.001) were significant predictors, indicating that cost, trust, and satisfaction directly influence attendants’ intentions to return to the facility. However, DDS (β = 0.008, *p* = 0.879) and WOM (β = −0.044, *p* = 0.334) did not show significant direct effects on RI, suggesting that their influence may be mediated through satisfaction. The overall graphical presentation of the path model is shown in Figure 2. The structural model demonstrated moderate explanatory and predictive power. The R^2^ values were 0.380 for perceived patient satisfaction and 0.477 for perceived revisit intention, indicating that the model explains 38% and 47.7% of the variance in these respective constructs. The average predictive relevance (Q^2^) across the model was 0.233, suggesting medium predictive capability, as values above 0.15 are considered indicative of moderate predictive relevance [33]. The model SRMR is 0.062 indicated acceptable fit. Bootstrapping with 5000 resamples confirmed path stability. Effect sizes (f^2^) varied across paths ranging from 0.022 to 0.211 indicating the small to medium effect [40] highlights; perceived patient satisfaction is the most influential construct in predicting perceived revisit intention, along with trust and cost.

### 3.5. Mediation Analysis

Mediation analysis, presented in Table 3, confirmed that patient satisfaction (PS) plays a significant mediating role between the exogenous constructs and revisit intention (RI). All indirect paths via PS were statistically significant; these results indicate complementary mediation, where both the direct and indirect effects are significant and aligned in direction. Clinically the mediation analysis suggests that satisfaction is a key lever for service retention.

### 3.6. Out-of-Sample Predictive Assessment

To further assess the robustness of our findings we use the PLSpredict, presented in Table S4, which demonstrated that all Q^2^_predict values were positive (Q^2^ > 0), indicating the predictive relevance of the model. Additionally, comparison between the prediction errors between PLS-SEM model and linear benchmark model (LM) showed largely comparable root mean squared error (RMSE) [30,41], with half of the indicators that showed lower prediction errors under the PLS model and the rest that showed only marginal difference, indicating acceptable out-of-sample predictive performance.

### 3.7. Multi-Group Analysis Across Facility Types

For the comparison of the structural relationship, multi-group analysis (MGA) was performed and presented in Table S5**.** The MGA result showed that most structural relationships did not significantly differ across facility types, while the effects of cost on perceived revisit intention (*p* = 0.033), perceived patient satisfaction on perceived revisit intention (*p* = 0.002), and perceived trust in healthcare providers on perceived patient satisfaction (*p* = 0.007) significantly differed between public and private facilities, indicating that affordability, trust and satisfaction play a comparative roles in shaping the revisit intention among the public dialysis facilities.

## 4. Discussion

The study revealed that adult attendants of dialysis patients in Dhaka expressed a strong intent to revisit the same dialysis facilities. Perceived revisit intention was significantly influenced by cost, perceived trust in healthcare providers, and perceived patient satisfaction. While dialysis service quality and word of mouth did not show direct effects on perceived revisit intention, both contributed indirectly by enhancing perceived patient satisfaction. Mediation analysis confirmed that perceived patient satisfaction plays a central role in linking service quality, trust, word of mouth, and cost to revisit behavior. These findings highlight the importance of trust, perceived value, and satisfaction in shaping loyalty among dialysis patient attendants in urban Bangladesh.

Trust in healthcare providers was a significant predictor of revisit intention. Studies across South and Southeast Asia have shown that when patients or their caregivers perceive providers as competent, respectful, and communicative, they are more likely to return for follow-up care and adhere to treatment plans [3,4,7,9,42]. In resource-constrained settings, trust also compensates for infrastructural limitations, making it a critical component of service retention [23,43].

In Bangladesh, where dialysis is largely financed out-of-pocket, affordability directly influences facility choice and continuity of care. Evidence from national assessments and regional studies confirms that high treatment costs often lead to delayed or discontinued dialysis, while facilities offering subsidized or tiered pricing structures report higher revisit rates [3,4,28,42]. Our findings support this pattern, suggesting that perceived cost fairness enhances both satisfaction and loyalty.

In our study, perceived patient satisfaction not only predicted revisit intention directly but also mediated the effects of service quality, trust, word of mouth, and cost, confirming its pivotal role in shaping loyalty among dialysis patient attendants. Prior studies have shown that satisfied patients and caregivers are more likely to recommend services, comply with treatment, and return for future care [21,35,43,44,45]. In our study, satisfaction not only predicted revisit intention directly but also mediated the effects of other service-related constructs, confirming its pivotal role in shaping loyalty.

Perceived dialysis service quality and word of mouth did not show significant direct effects on revisit intention. These findings suggest that both constructs influence revisit intention primarily through perceived patient satisfaction rather than direct behavioral responses. This mediation contrasts with findings from other LMIC contexts, where both constructs have been shown to influence patient retention [43,44,46]. The discrepancy may reflect differences in how attendants rather than patients evaluate service quality, or it may suggest that these factors exert their influence primarily through satisfaction. Additionally, in Dhaka’s urban context, where proximity and familiarity often shape healthcare decisions, word of mouth may exert a weaker influence on revisit behavior than previously reported [47]. These results suggest that while service quality and WOM are important, their effects on loyalty are likely mediated through overall satisfaction.

This study included multiple dialysis centers in Dhaka, improving contextual relevance. Moreover, the sample characteristics were compared with the national dialysis statistics and show similar distribution, though generalizability remains limited [3]. The use of a localized, literature-informed tool and trained staff helped ensure data quality. PLS-SEM allows detailed analysis of complex relationships. However, several limitations should be acknowledged. The reliance on self-reported data may introduce social desirability bias. The purposive sampling of facilities limits generalizability and may introduce selection bias. Additionally, the exclusion of attendants of inpatients and those with severe medical conditions may have omitted perspectives from more vulnerable caregiving contexts. The data did not meet the assumption of multivariate normality, which, although permissible in PLS-SEM, may affect the generalizability of parametric interpretations. The study focused solely on attendants’ perspectives, which may differ from patients’ own experiences and decision-making processes. Additionally, when it comes to trust in facility and staff, which is built over time, or the word of mouth, our inclusion criteria did not account for the duration of the taking of the dialysis a specific facility, which influences the trust issues. Future studies should consider these issues. Finally, this is a cross-sectional study, and the findings should be interpreted as an association rather than a causal relationship.

## 5. Conclusions

This study examined the factors influencing revisit intention among adult attendants of dialysis patients in Dhaka. The findings indicate that cost, trust in healthcare providers, and patient satisfaction significantly contribute to revisiting behavior, with patient satisfaction playing a central mediating role. While dialysis service quality and word of mouth did not show direct effects on revisit intention, both contributed indirectly through their influence on satisfaction. These results suggest that enhancing trust, perceived value, and satisfaction may be effective strategies for improving service retention in urban dialysis settings. Importantly, this study was conducted during the dialysis sessions; in the context of Bangladesh it is common to present a caregiver during the treatment session, but the perspective of the caregiver is also very important in the context of Bangladesh as suggested by the findings of our study. However, the interpretation of recommendation is framed cautiously, emphasizing affordability trust and satisfaction as empirically supported drivers of revisit intention rather than prescriptive causal determinants. The non-significant direct effects of service quality and word of mouth highlight the need for further investigation into how these constructs operate in caregiver decision-making contexts. Additionally, the government could consider subsidy models, trust-building programs, and targeted service quality improvements.

## Figures and Tables

**Table 1:** Socio-Demographic Characteristics of the Respondents.

Variable	Items	Frequency	Percentage
Gender	Male	239	59.9%
	Female	160	40.1%
Educational	Primary/secondary/higher secondary	174	43.6%
	Diploma and equivalent	159	39.8%
	Bachelor/Master/decorated degree	46	11.5%
	Others	20	5.0%
Household Income †	Below 20,000	69	17.3%
	20,001–40,000	23	5.8%
	40,001–60,000	132	33.1%
	60,001–80,000	56	14.0%
	80,001–100,000	56	14.0%
	100,001–Above	63	15.8%
Patients Age Group	18–30	32	8.0%
	31–44	56	14.0%
	45–49	129	32.3%
	50–59	42	10.6%
	60–64	124	31.1%
	65 and above	16	4.0%
Residence	Urban	337	84.5%
	Rural	62	15.5%
Reason For Choosing the Dialysis Center	Located Nearby	242	60.7%
	Doctor Referral	99	24.8%
	Others factor	58	14.5%

Note. † Currency values are presented in Bangladeshi Taka (BDT).

**Table 2:** Hypothesis Testing for Path Coefficient.

Hypothesis	Coefficient	t-Values	95% CI	*p*-Value	Decision	Q^2^	R^2^	f^2^
CO -> PS	0.294	4.441	0.163, 0.409	<0.001	Accepted	0.187	0.380	0.074
DDS -> PS	0.152	3.033	0.038, 0.238	0.003	Accepted			0.022
THP -> PS	0.229	4.317	0.126, 0.332	<0.001	Accepted			0.057
WOM -> PS	0.103	2.128	0.013, 0.192	0.034	Accepted			0.024
CO -> RI	0.167	2.936	0.061, 0.281	0.003	Accepted	0.279	0.477	0.026
DDS -> RI	0.008	0.152	−0.099, 0.102	0.879	Rejected			0.000
PS -> RI	0.422	7.760	0.321, 0.523	<0.001	Accepted			0.211
THP -> RI	0.252	5.379	0.168, 0.344	<0.001	Accepted			0.078
WOM -> RI	−0.044	0.966	−0.136, 0.037	0.334	Rejected			0.003

Note. DDS = dialysis delivery service; THP = trust in healthcare providers; WOM = word of mouth; CO = cost; PS = patient satisfaction; RI = revisit intention; R^2^ indicates the proportion of variance explained by the exogenous constructs. Q^2^ reflects the model’s predictive relevance, and f^2^ represents the effect size; CI = Confidence Interval.

**Table 3:** Hypothesis Testing for Mediation Effect.

Hypothesis	Coefficient	t-Values	95% CI	*p*-Value	Mediation Type
THP -> PS -> RI	0.097	4.386	0.055, 0.140	<0.001	Complementary
WOM -> PS -> RI	0.044	2.103	0.005, 0.084	0.036	Complementary
DDS -> PS -> RI	0.064	2.641	0.019, 0.114	0.009	Complementary
CO -> PS -> RI	0.124	3.624	0.059, 0.195	<0.001	Complementary

Note. DDS = dialysis delivery service; THP = trust in healthcare providers; WOM = word of mouth; CO = cost; PS = patient satisfaction; RI = revisit intention; CI = Confidence Interval.

## Data Availability

The data supporting the findings of this study are available from the corresponding author upon reasonable request. Due to ethical considerations and participant confidentiality, the dataset is not publicly shared.

## Supplementary Materials

The following supporting information can be downloaded at: https://www.mdpi.com/article/10.3390/ijerph23060769/s1, Table S1: Survey Instrument; Table S2. Reliability Analysis; Table S3. Discriminate Validity; Table S4. Assessment of Out-Of-Sample Predictive Performance; Table S5. Multi-Group Analysis (MGA) Across Public and Private Dialysis Facilities.

## References

[1] Haque W.M.M.U., Hossain D., Amin M.F., Samad T., Mohsena M., Habib S.H., Rahim M.A., Alam M., Billah M.M., Mehfuz-E-Khoda M. (2024). Diabetic Kidney Disease in Bangladesh: A Cross-Sectional Study on Screening, Treatment and Prevention Practice. IMC J. Med. Sci..

[2] Wainstein M., Tiv S., Arruebo S., Caskey F.J., Damster S., Donner J.-A., Gouda Z., Jha V., Levin A., Nangaku M. (2025). Global Policy and Advocacy Initiatives for Improving Kidney Care: Report from the 2023 International Society of Nephrology Global Kidney Health Atlas. Kidney360.

[3] Ripon M.S.H., Ahmed S., Rahman T., Rashid H.-U., Karupaiah T., Khosla P., Daud Z.A.M., Arefin S.U.Z., Osmani A.S. (2023). Dialysis Capacity and Nutrition Care Across Bangladesh: A Situational Assessment. PLoS ONE.

[4] Rashid H.U., Alam M.R., Khanam A., Rahman M.M., Ahmed S., Mostafi M., Arefin S.U.Z., Kashem T.S., Begum N.A.S., Alam K.S., Moura-Neto J.A., Divino-Filho J.C., Ronco C. (2021). Nephrology in Bangladesh. Nephrology Worldwide.

[5] Thurlow J.S., Joshi M., Yan G., Norris K.C., Agodoa L.Y., Yuan C.M., Nee R. (2021). Global Epidemiology of End-Stage Kidney Disease and Disparities in Kidney Replacement Therapy. Am. J. Nephrol..

[6] Cohen-Hagai K., Kitani A., Benchetrit S., Erez D., Alon A., Wilf-Miron R., Saban M. (2024). The Patient’s Perspective: Does It Align with Dialysis Adequacy?. Kidney360.

[7] Chowdhury M.H., Islam M.A., Islam F., Mobarak R., Ghosh K. (2024). Chronic Kidney Disease in Children of A Paediatric Critical Care Nephrology & Dialysis Department: A Study in Bangladesh Shishu (Children) Hospital & Institute, Bangladesh. IOSR J. Dent. Med. Sci..

[8] Mahbub M.R., Shadat M.A., Goni M.O., Chowdhury S. (2024). Chronic Kidney Disease and Risk Factors Among Type 2 Diabetic Patients in Selected Hospitals in Dhaka, Bangladesh. Health Sci. Q..

[9] Woo S., Choi M. (2021). Medical Service Quality, Patient Satisfaction and Intent to Revisit: Case Study of Public Hub Hospitals in the Republic of Korea. PLoS ONE.

[10] Hosseinzadeh M., Pouladzadeh M., Eskandari A. (2024). Assessment of Healthcare Service Quality and Patient Satisfaction Using the SERVQUAL Questionnaire in Khuzestan Province During 2022–2023. Jundishapur J. Chronic Dis. Care.

[11] Akanyako J. (2024). Does Patient Satisfaction and Trust Matter in the Relationship Between Service Quality and Patient Loyalty in the Ghanaian Health Sector?. Afr. J. Empir. Res..

[12] Vernanda N., Wardi Y. (2025). The Influence of Service Quality on Word of Mouth Through Patient Satisfaction and Trust as Mediating Variables at Balad Medical Center Clinic, Pariaman. J. Ris. Multidisiplin Edukasi.

[13] Wider W., Tan F.P., Tan Y.P., Lin J., Fauzi M.A., Wong L.S., Tanucan J.C.M., Hossain S.F.A. (2024). Service Quality (SERVQUAL) Model in Private Higher Education Institutions: A Bibliometric Analysis of Past, Present, and Future Prospects. Soc. Sci. Humanit. Open.

[14] Greene J., Samuel-Jakubos H. (2021). Building Patient Trust in Hospitals: A Combination of Hospital-Related Factors and Health Care Clinician Behaviors. Jt. Comm. J. Qual. Patient Saf..

[15] Pervaiz S., Javed U., Rajput A., Shafique S., Tasneem R. (2024). Examining How and Why Service Quality Fosters Patients’ Revisit Intentions: Evidence from Pakistan. Int. J. Progn. Health Manag..

[16] Patil B., Morabiya M., Patidar N. (2024). A Study Emphasizing That Healthcare Service Quality Is Positively Related with Patient Satisfaction and Patient Loyalty. Int. Res. J. Mod. Eng. Technol. Sci..

[17] Liu J. (2024). Influence of Service Quality and Customer Perceived Value on Customer Loyalty with Customer Satisfaction as a Moderating Factor: A Study Based on Private Elderly Care Services in China. J. Infrastruct. Policy Dev..

[18] Palad J.T. (2024). Service Delivery of Hemodialysis Centers among Public Hospitals in Region III: An Evaluation. Int. Res. Innov. J..

[19] Zerbinati L., Guerzoni F., Napoli N., Preti A., Esposito P., Caruso R., Bulighin F., Storari A., Grassi L., Battaglia Y. (2023). Psychosocial Determinants of Healthcare Use Costs in Kidney Transplant Recipients. Front. Public Health.

[20] Dusak P.K. (2025). The Effects of Service Quality and Additional Factors to Electronic Word of Mouth and Revisit Intention at Dental Clinics Mediated by Patient Satisfaction. Eduvest.

[21] Rum M.R., Nursanty O.E. (2024). Patient Satisfaction with the Quality of Nursing Care in the Outpatient Department of the Hospital. J. Ilm. Ilmu Keperawatan Indones..

[22] Sekar H., Tan P.H.P. (2024). The Relationship between Hospital Service Quality and Patient Trust Is Mediated by Patient Satisfaction in Patient Loyalty at Hospital X. J. Syntax Lit..

[23] Hidayat A.S., Handoyo S.E. (2025). The Effect of Service Quality and Patient Satisfaction on Revisit Intentions of Inpatients at Ciawi Regional Public Hospital Mediated by Patient Trust. J. Humanit. Soc. Sci. Stud..

[24] Kim M.-G. (2023). Optimizing Patient Outcomes in Dialysis Patients: The Significance of Dialysis Specialists. Kidney Res. Clin. Pract..

[25] Priyanto E.B., Rahayuningsih E., Anggiani S. (2025). The Impact of Hospital Service Technology and Service Quality on Patient Revisit Intention: Mediating Role of Patient Experience. Parad. J. Ilmu Ekon..

[26] Dialysis Patient Satisfaction Survey (DPSS). https://www.rand.org/health/surveys/dpss.html.

[27] Park S., Kim H.-K., Choi M., Lee M. (2021). Factors Affecting Revisit Intention for Medical Services at Dental Clinics. PLoS ONE.

[28] Sanabria-Arenas M., Marín J.T., Certuche-Quintana M.C., Sánchez-Pedraza R. (2017). Validation of an Instrument for Measuring Satisfaction of Patients Undergoing Hemodialysis. BMC Health Serv. Res..

[29] Hair J.F., Risher J.J., Sarstedt M., Ringle C.M. (2019). When to Use and How to Report the Results of PLS-SEM. Eur. Bus. Rev..

[30] Hair J., Alamer A. (2022). Partial Least Squares Structural Equation Modeling (PLS-SEM) in Second Language and Education Research: Guidelines Using an Applied Example. Res. Methods Appl. Linguist..

[31] Hair J.F., Ringle C.M., Sarstedt M. (2011). PLS-SEM: Indeed a Silver Bullet. J. Mark. Theory Pract..

[32] Cronin J.J., Taylor S.A. (1992). Measuring Service Quality: A Reexamination and Extension. J. Mark..

[33] Chin W.W., Esposito Vinzi V., Chin W.W., Henseler J., Wang H. (2010). How to Write up and Report PLS Analyses. Handbook of Partial Least Squares.

[34] Mayer R.C., Davis J.H., Schoorman F.D. (1995). An Integrative Model of Organizational Trust. Acad. Manag. Rev..

[35] Islam S.B., Bhat S.A., Darzi M.A., Khursheed S.O. (2025). Investigating Patient Revisit Intention in Community Health Centres: A Mediational Study. Int. J. Progn. Health Manag..

[36] Ajzen I. (1991). The Theory of Planned Behavior. Organ. Behav. Hum. Decis. Process..

[37] Kock N. (2015). Common Method Bias in PLS-SEM: A Full Collinearity Assessment Approach. Int. J. e-Collab..

[38] Fornell C., Larcker D.F. (1981). Structural Equation Models with Unobservable Variables and Measurement Error: Algebra and Statistics. J. Mark. Res..

[39] Henseler J., Ringle C.M., Sarstedt M. (2015). A New Criterion for Assessing Discriminant Validity in Variance-Based Structural Equation Modeling. J. Acad. Mark. Sci..

[40] Cohen J. (2013). Statistical Power Analysis for the Behavioral Sciences.

[41] Shmueli G., Sarstedt M., Hair J.F., Cheah J.-H., Ting H., Vaithilingam S., Ringle C.M. (2019). Predictive Model Assessment in PLS-SEM: Guidelines for Using PLSpredict. Eur. J. Mark..

[42] Kar S., Islam M.F. (2023). Global Dialysis Perspective: Bangladesh. Kidney360.

[43] Yuniarti Y., Hidayat A. (2021). The Analysis of Patients’ Revisits Intention Factors in Sub-Urban Hospital. Int. J. Res. Bus. Soc. Sci..

[44] Sundram S., Tambvekar S.E., Sekar S., Tiwari S.K., Gopinathan R. (2022). The Effect of Service Quality on Patient Loyalty Mediated by Patient Satisfaction. J. Pharm. Negat. Results.

[45] Januarko M.U., Hapsari N.P., Sofwan I. (2023). Analysis of Retreatment Intention Influenced Quality of Service, Patient Trust, Healthy Culture, Dimediation of Patient Satisfaction at Betawi Hospital, North Jakarta. Maj. Ilm. Bijak.

[46] Choi K.-S., Cho W.-H., Lee S., Lee H., Kim C. (2004). The Relationships among Quality, Value, Satisfaction and Behavioral Intention in Health Care Provider Choice. J. Bus. Res..

[47] Shahabuddin A.M., Rahman M.T., Ahsan S.M.H., Islam M.S., Akter K. (2024). Antecedents of Revisit Intentions on Hospital Choice in the Developing Country: A SEM Analysis. Am. Int. J. Soc. Sci. Res..

