# Supplementary material for: Exploring Facility Revisit Intentions Among the Kidney Dialysis Patient’s Attendance: Evidence from a Cross-Sectional Study in Dhaka, Bangladesh
Source: Int J Environ Res Public Health. 2026 Jun 7;23(6):769. doi: 10.3390/ijerph23060769 (PMC13299428; doi:10.3390/ijerph23060769)
Supplement: Supplementary file 1 [file ijerph-23-00769-s001.zip › ijerph-4194935-supplementary.pdf]

Table S1. Survey Instrument

Derived Scale Items	Code	Authors
**Perceived Dialysis Delivery Service (DDS)**		[9,26–28]
I perceive that the process for making dialysis appointments works quickly and easily.	DDS_1	
I perceive that the dialysis center is clean and follows appropriate hygiene standards.	DDS_2	
I perceive that the staff at the dialysis center are friendly and willing to help patients and attendants.	DDS_3	
I perceive that the facilities and equipment used for dialysis treatment are clean and function properly.	DDS_4	
Overall, I am satisfied with the care and service provided by the dialysis center.	DDS_5	
**Perceived Trust in Healthcare Providers (THP)**		
I believe that healthcare providers make treatment decisions that are in the best interest of the patient.	THP_1	
I perceive that the patient feels comfortable communicating openly with healthcare providers about health-related concerns.	THP_2	
I believe that healthcare providers are competent and knowledgeable in delivering dialysis care.	THP_3	
I trust that the healthcare providers maintain patient privacy and confidentiality appropriately.	THP_4	
I have confidence in the guidance and recommendations provided by the healthcare professionals.	THP_5	
**Perceived Word of Mouth (WOM)**		
The quality of dialysis services influences my conversations with others about this dialysis center.	WOM_1	
I often recommend this dialysis center to others based on my experiences and observations.	WOM_2	
Positive experiences with this dialysis center encourage me to share my opinions with friends and family.	WOM_3	
Hearing positive experiences from others increases my trust in this dialysis service.	WOM_4	
**Cost (CO)**		
I consider the cost of the dialysis service to be reasonable.	CO_1	
Considering the quality of care provided, I believe the cost of dialysis services is justified.	CO_2	
I am concerned about the financial burden associated with dialysis treatment.	CO_3	
I am satisfied with the transparency of costs and billing procedures related to dialysis services.	CO_4	

**Perceived Patient Satisfaction (PS)**	
I am satisfied with the overall quality of care provided by the dialysis service.	PS_1
I perceive that the staff at the dialysis center are attentive to patient needs.	PS_2
I perceive that the patient receives dialysis treatment in a comfortable and safe environment.	PS_3
The dialysis service meets my expectations regarding quality healthcare delivery.	PS_4
**Perceived Re-visit Intention (RI)**	
I intend to continue utilizing this dialysis service for the patient's future treatment needs.	RI_1
I would choose this dialysis service again for the patient if needed.	RI_2
I plan to continue using this dialysis service for the patient as long as necessary.	RI_3
I am likely to recommend this dialysis service to others who require similar care.	RI_4

Table S2. Reliability Analysis

Variables	Cronbach's Alpha	rho_A	Composite Reliability	AVE	VIF
CO	0.733	0.738	0.832	0.554	1.883
DDS	0.796	0.809	0.863	0.563	1.679
PS	0.705	0.702	0.815	0.525	1.614
RI	0.784	0.785	0.861	0.607	-
THP	0.781	0.793	0.852	0.537	1.478
WOM	0.751	0.799	0.846	0.587	1.270

Note. DDS = Dialysis Delivery Service; THP = Trust in Healthcare Providers; WOM = Word of Mouth; CO = Cost; PS = Patient Satisfaction; RI = Re-visit Intention; AVE = Average Variance Extracted; VIF = Variance Inflation Factor; rho_A = an internal consistency reliability measure for reflective constructs; AVE > 0.50 indicates convergent validity[29,38]; VIF < 3.3 indicates absence of problematic collinearity[37]

Table S3. Discriminate Validity

Variable	CO	DDS	PS	RI	THP	WOM
CO_1	0.761	0.468	0.479	0.430	0.451	0.532
CO_2	0.756	0.485	0.403	0.399	0.367	0.292
CO_3	0.731	0.386	0.319	0.353	0.323	0.253
CO_4	0.727	0.411	0.408	0.335	0.377	0.218
DDS_1	0.446	0.794	0.372	0.247	0.321	0.209
DDS_2	0.455	0.864	0.385	0.341	0.390	0.259
DDS_3	0.475	0.854	0.390	0.323	0.401	0.268
DDS_4	0.453	0.602	0.363	0.364	0.373	0.289
DDS_5	0.364	0.590	0.230	0.266	0.367	0.235
PS_1	0.408	0.296	0.696	0.467	0.406	0.222
PS_2	0.378	0.271	0.676	0.359	0.314	0.332
PS_3	0.438	0.430	0.775	0.467	0.335	0.292
PS_4	0.364	0.365	0.746	0.505	0.350	0.190
RI_1	0.381	0.239	0.506	0.753	0.372	0.187
RI_2	0.454	0.358	0.488	0.779	0.387	0.235
RI_3	0.345	0.370	0.519	0.808	0.464	0.234
RI_4	0.424	0.330	0.432	0.776	0.441	0.140
THP_1	0.370	0.369	0.348	0.402	0.808	0.254
THP_2	0.272	0.258	0.315	0.380	0.618	0.124
THP_3	0.428	0.373	0.364	0.370	0.695	0.217
THP_4	0.366	0.406	0.305	0.323	0.696	0.181
THP_5	0.440	0.410	0.428	0.465	0.826	0.262
WOM_1	0.443	0.382	0.311	0.269	0.306	0.899
WOM_2	0.321	0.173	0.221	0.182	0.185	0.589
WOM_3	0.199	0.130	0.257	0.079	0.102	0.633
WOM_4	0.375	0.296	0.289	0.218	0.247	0.891
**Fornell-Larcker criterion**						
CO	0.744					
DDS	0.592	0.751				
PS	0.548	0.474	0.724			
RI	0.514	0.417	0.625	0.779		
THP	0.516	0.496	0.485	0.535	0.733	
WOM	0.451	0.339	0.353	0.256	0.288	0.766
**Heterotrait-Monotrait Ratio (HTMT)**						
CO						
DDS	0.767					
PS	0.755	0.624				
RI	0.673	0.524	0.839			
THP	0.671	0.635	0.653	0.679		
WOM	0.576	0.419	0.499	0.321	0.358	

Note. DDS = Dialysis Delivery Service; THP = Trust in Healthcare Providers; WOM = Word of Mouth; CO = Cost; PS = Patient Satisfaction; RI = Re-visit Intention; HTMT < 0.85 indicates discriminant validity[29,39]

Table S4. Assessment of Out-Of-Sample Predictive Performance

Indicator	RMSE (PLS)	RMSE (LM)	Q^2_predict	Interpretation
PS_1	0.653	0.649	0.196	Comparable
PS_2	0.628	0.624	0.164	Comparable
PS_3	0.593	0.609	0.226	PLS better
PS_4	0.563	0.573	0.166	PLS better
RI_1	0.584	0.597	0.169	PLS better
RI_2	0.583	0.591	0.223	PLS better
RI_4	0.624	0.624	0.235	Comparable
RI_3	0.659	0.651	0.216	Comparable

Note. RMSE = Root Mean Squared Error

Table S5. Multi-Group Analysis (MGA) Across Public and Private Dialysis Facilities

Inputs	Public Hospital		Private Hospital		Difference	MGA p-Value	Interpretation
	beta	95% CI	Beta	95% CI			
CO -> PS	0.244	0.079, 0.397	0.339	0.153, 0.485	-0.095	0.778	No significant group difference
CO -> RI	0.261	0.112, 0.376	0.037	-0.153, 0.24	0.224	0.033	Significant difference; stronger in public facilities
DDS -> PS	0.147	0.009, 0.268	0.180	0.026, 0.364	-0.033	0.610	No significant group difference
DDS -> RI	-0.063	-0.17, 0.053	0.290	0.098, 0.449	-0.353	1.000	Group difference not statistically supported
PS -> RI	0.540	0.395, 0.677	0.228	0.036, 0.39	0.313	0.002	Significant difference; stronger in public facilities
THP -> PS	0.326	0.19, 0.452	0.074	-0.1, 0.219	0.252	0.007	Significant difference; stronger in public facilities
THP -> RI	0.177	0.035, 0.284	0.226	0.064, 0.374	-0.049	0.691	No significant group difference
WOM -> PS	0.052	-0.084, 0.161	0.201	0.053, 0.362	-0.149	0.934	No significant group difference
WOM -> RI	-0.092	-0.178, 0.014	0.015	-0.134, 0.159	-0.107	0.885	No significant group difference

Note. CI = Confidence Interval; MGA = Multi-Group Analysis
